# Total Flavonoids of *Crocus sativus* Petals Release *tert*-Butyl Hydroperoxide-Induced Oxidative Stress in BRL-3A Cells

**DOI:** 10.1155/2021/5453047

**Published:** 2021-06-05

**Authors:** Hong Ye, Juan Luo, Dongmei Hu, Shuting Yang, Aolai Zhang, Yanxia Qiu, Xiaona Ma, Jing Wang, Jing Hou, Jie Bai

**Affiliations:** Key Laboratory of Bio-Resource and Eco-Environment of Ministry of Education, College of Life Sciences, Sichuan University, Chengdu, 610065 Sichuan, China

## Abstract

Antioxidant and hepatoprotective activities in vitro of saffron petals were examined in this study for better utilizing saffron (*Crocus sativus* L.) biowaste. Using the DPPH and ABTS radical scavenging method, we compared the antioxidant activity and the content of total flavonoid extracts from petals (TFESP), stamens (TFESS), and both saffron petals and stamens (TFEMS). The results showed that the antioxidant capacity and the flavonoid content of TFESP were higher than those of TFESS and TFEMS. Then, the hepatoprotective activity of TFESP was determined, and the silymarin was used as a positive control. The main components of TFESP were analysed by ultrahigh performance liquid chromatography (UPLC) photodiode array (PDA)/mass spectrometry (MS) and nuclear magnetic resonance (NMR). The result showed that (1) TFESP could release oxidative liver injury induced by *tert*-butyl hydroperoxide (t-BHP). (2) TFESP could reduce the accumulation of reactive oxygen species (ROS); enhance the activity of superoxide dismutase (SOD), catalase (CAT), and glutathione (GSH); and then improve the total antioxidant capacity (T-AOC) in BRL-3A cells. (3) TFESP could enhance the expression of B-cell lymphoma-2 (*BCL-2*) and decrease the expression of *caspase-3* and *caspase-9*; increase the expression of Kelch-like ECH-associated protein-1 (*Keap-1*), *nuclear factor, erythroid 2-related factor 2* (*Nrf2*), superoxide dismutase, and heme oxygenase 1 (*HO-1*); and downregulate inducible nitric oxide synthase (*INOS*), interleukin-6 (*IL-6*), and nuclear factor kappa B-9 (*NF-κB-9*). (4) The main hepatoprotective component of TFESP was identified as kaempferol-3-o-sophoroside. The mechanism may be that kaempferol-3-o-sophoroside can protect t-BHP-induced cell injury by regulating the expression of antioxidant, antiapoptotic, and anti-inflammatory genes. Thus, saffron petals are a potential hepatoprotective resource worthy of development.

## 1. Introduction

Saffron (*Crocus sativus* L.), a perennial bulbous herb of the genus *Crocus* in the Iridaceae family, has been used as an ingredient in medicine, tea, and cooking seasonings for ages [1, 2]. Many studies have shown that saffron has numerous beneficial effects on human health, including antioxidant, anti-inflammation [3], anticancer [4], antidepressant [5], antihypertensive [6], anticardiovascular disease [7], and hepatoprotective activity [8]. However, these effects essentially come from the stigmas of saffron. The production of saffron stigma also generates a lot of floral biowaste, especially petals. Although recent studies showed a variety of their benefits in practice, petals are usually discarded, leading to unnecessary waste of natural resources [9].

Saffron petals contain a variety of compounds, such as minerals, anthocyanins, flavonoids, glycosides, and alkaloids [10, 11]. As such, they may have rich pharmacological functions [12, 13], including hepatoprotective activity. In recent years, studies on extracts from saffron flowers have found that its main component is glycosylated flavonoids. At present, flavonoids of saffron petals have been proven to have antioxidant activity and anti-inflammatory and regulating blood pressure effects, but their protective effects on the liver have been rarely studied. Iranshahi's research indicated that extracts of stigma and petals have hepatoprotective activity [14]. In addition, many studies have shown that stigma has hepatoprotective activity. Omidi et al. indicated that the alcoholic extract of the petals has hepatoprotective activity by detecting ALT, AST, and total protein of male Wistar rats [15]. However, it is not further explained whether petals have hepatoprotective activity on oxidative liver injury. Therefore, it is still unclear whether the specific organ of saffron petals has hepatoprotective activity and what is the material basis for liver protection.

The compound, *tert*-butyl hydroperoxide (t-BHP), has been routinely used in the establishment of in vitro oxidative stress damage models because it can induce acute oxidative stress in vivo and in vitro, which in turn causes acute liver injury [16]. More specifically, t-BHP can induce excess of peroxyl and alkoxy groups in the free radical intermediates in liver cells, resulting in excess ROS, which produces acute oxidative stress in the cells and causes acute liver damage. Besides, t-BHP can convert reduced glutathione (GSH) in the body into oxidized glutathione (GS-SG), thereby causing in vivo reduction of reduced glutathione [17]. ROS is mainly eliminated by GSH, and the transitional consumption of GSH can reduce its ability to scavenge ROS, which will increase oxidative stress in the body [18].

Other studies have shown that silymarin has great potential for clinical application as it can effectively fight against toxic hepatitis [19], fatty liver, ischemic injury, and radiation toxicity due to its antioxidation, antilipid peroxidation, antifibrosis, anti-inflammation, immune regulation, and liver regeneration [20]. Silymarin has been considered as a positive control compound for hepatoprotective effects in drug research, which is also adopted in this study.

The purpose of this study was to investigate the hepatoprotective effect of total flavonoid extracts of saffron petals against acute hepatic injury induced by t-BHP and reveal its mechanism.

## 2. Materials and Methods

### 2.1. Materials

The *C. sativus* was grown in Anji County, Zhejiang Province, China, and was provided by Lvshengxuan Biotechnology Company. The normal rat liver cell line (BRL-3A) was taken from the American Type Culture Collection (ATCC, CRL-1442; Manassas, VA, USA).

Dulbecco's modified Eagle's medium (DMEM) and fetal bovine serum (FBS) for cell culture and chromatographically pure acetonitrile and methanol for UPLC were purchased from Thermo Fisher Scientific (Waltham, MA, USA). Analytical pure ethanol and petroleum were purchased from the Chengdu Kelong Chemical Reagent Factory (Sichuan, China). The AB-8 macroporous resin was purchased from Tianjin Nankai University Chemical Factory (Tianjin, China). 2,2′-Azino-bis (3-ethylbenzthiazoline-6-sulfonic acid) (ABTS), 2,2-diphenyl-1-picrylhydrazyl (DPPH), and vitamin C (Vc) were purchased from Sigma-Aldrich Chemical Co. (St. Louis, MO, USA). Cell counting kit 8 (CCK-8) used to determine the viability of cells was purchased from KeyGen Biotech (Jiangsu, China). Alanine aminotransferase (ALT), aspartate transaminase (AST), catalase (CAT) enzyme activity bicinchoninic acid (BCA) protein quantification, glutathione (GSH), lactate dehydrogenase (LDH), superoxide dismutase (SOD), cellular ROS detection assay kits, and total antioxidant capacity (T-AOC) were purchased from Jiancheng Bioengineering Company (Nanjing, China). 4′,6-Diamidino-2-phenylindole (DAPI) for staining of living cells and radioimmunoprecipitation assay (RIPA) buffer for cell lysis were purchased from SolarBio (Beijing, China) and NCM Biotech (Suzhou, China), respectively. TRIzol total RNA extraction kit, reverse transcription kit, and RT-qPCR kit were purchased from Aikerui Biotech (Hunan, China). All contractions used in this article are listed in the supplementary material (Table S2.).

### 2.2. Extraction Preparation

The 50 g dried powder of saffron petals (SP), stamens (SS), and mixture of petals and stamens (MPS) was degreased twice with 2.5 L petroleum ether under ultrasonication at 59 Hz and 55°C for 30 min, respectively. After degreasing, the SP, SS, and MPS were dried at a constant temperature of 45°C. Degreased SP, SS, and MPS were extracted three times with 250 mL 70% ethanol under ultrasonication at 59 Hz and 55°C for 40 min. The filtrates were placed in a spin-dried evaporator to remove the organic solvent and lyophilized with a freeze desiccant. The crude extracts were dissolved in distilled water and then were loaded into AB-8 macroporous resin. The AB-8 macroporous resin was eluted with ultrapure water and absolute ethanol. The absolute ethanol eluents were collected and dried in a rotary vacuum evaporator. Acquired total flavonoid extracts from saffron petals (TFESP), stamens (TFESS), and mixture of petals and stamens (TFEMS) were dissolved in 50% (*v*/*v*) ethanol for antioxidant activity determination. TFESP, TFESS, and TFEMS were dissolved in serum-free medium cell experiments.

### 2.3. In Vitro Antioxidant Activity of TFESP

As described previously [13], the total flavonoid content (TFC) of TFESP was tested by colorimetry, with the linear equation method (*y* = 0.4195*x* + 0.0563; *R*^2^ = 0.9993). The content of the total flavonoid was expressed as rutin equivalents (RE). DPPH and ABTS radical removing activities were determined using a method with Vc as positive control, which was described previously and represented by half-maximal inhibitory concentrations (IC_50_, the concentrations required to remove 50% of the radicals) [13].

### 2.4. Cell Culture

The normal rat liver cell line (BRL-3A) was cultured with DMEM containing 10% FBS, 100 IU/mL penicillin, and 100 IU/mL streptomycin incubated at 37°C with a 5% CO_2_ atmosphere.

### 2.5. TFESP Toxicity Test Assay

CCK-8 was chosen for evaluating TFESP toxicity on the BRL-3A cell. The cells were cultured with 96-well plates at a density of 1 × 10^5^ cells/mL for reaching the logarithmic phase. At the 50~60% confluence, they were treated with various concentrations of TFESP for one day, followed by incubation in 10% CCK-8 solution for 1 h at 37°C. A microplate reader was used to measure the absorbance at 450 nm. The cells of the blank control were cultured with complete untreated medium. Each group had five wells, and the experiment was repeated more than three times.

### 2.6. Protective Effects of TFESP against t-BHP-Induced Damage

96-well plates and 6-well plates were seeded with BRL-3A cells at the logarithmic phase at a density of 1 × 10^4^ and 2.6 × 10^5^ cells/well, respectively. When the cells' density reached 50~60% confluence, they were incubated with 0, 25, 50, and 100 *μ*g/mL TFESP (control, low, middle, and high concentrations) and 20 *μ*g/mL silymarin (positive control) for 24 h, respectively. Subsequently, some cells were untreated, while other cells were incubated with 100 *μ*M t-BHP for 2 h. The survival rate of cells was detected by adding 10 *μ*L CCK-8 to each well in the 96-well plates, and then, the plate was placed in a 37°C thermostat for 1 h. And then, the absorbance of different samples at 450 nm was measured. The cells in the 6-well plate were treated with various concentration drugs. Then, the cells were washed twice with PBS, collected, and centrifuged. At last, the apoptosis rate and some other indicators of the cells were tested.

### 2.7. Measurement of Intracellular ROS

2′,7′-Dichlorodihydrofluorescein diacetate (DCFH-DA) was chosen to evaluate intracellular ROS levels according to previous reports [21]. In a few words, after cells in a 6-well plate were treated with t-BHP, PBS was chosen to wash the cells, and then, 5 *μ*M DCFH-DA was added into the cells. After dark treatment for 30 min, the cells were observed at a great lick under a fluorescence microscope (Olympus IX71, Olympus Corp., Tokyo, Japan) and photographed. Cells in another 6-well plate were gathered, dyed, and analysed by a BD FACSCalibur™ flow cytometer (BD Biosciences, Franklin Lakes, NJ, USA).

### 2.8. Real-Time Quantitative PCR (RT-qPCR) Analysis of Genes Related to Apoptosis

500 *μ*L TRIzol reagent was added to each well of a 6-well plate after the cells in it were treated with different drugs, and the total RNA was extracted from the cells according to the manufacturer's instructions. An agarose gel was used to visualize and assess the integrity of RNA. The RNA was reverse transcribed to cDNA by a reverse transcription (RT) kit. The primer sequences used in the RT-qPCR assay were all included in the supplemental material (Table S1). The real-time quantitative polymerase chain reaction (RT-qPCR) was used to determine gene expression with the real-time SYBR Green method by a Bio-Rad CFX96 thermocycler (Bio-Rad Laboratories, Hercules, CA, USA). Glyceraldehyde 3-phosphate dehydrogenase (GAPDH) was chosen as the internal reference gene to convert the CT to relative expression with the 2^-*ΔΔ*Ct^ data analysis method.

### 2.9. Measurement of Key Enzyme Level in Cell Supernatants

The levels of key enzyme (ALT, AST, and LDH) activities contained in the cell supernatants were detected by kits on request of commercial instructions [22]. Absorbances of the samples were tested by a microplate reader, and the activities of some key enzymes in cell supernatants (AST, ALT, and LDH) were shown as units per litre (U/L).

### 2.10. Determination of Antioxidant Activities

#### 2.10.1. Measurement of Antioxidant Activity Levels

The antioxidant levels of antioxidant activities of CAT, GSH, SOD, and T-AOC were determined as commercial test kit instructions [23]. Absorbances were tested by a microplate reader.

#### 2.10.2. RT-qPCR Analysis of Genes Related to Antioxidant Activities

The procedure adopted here was the same as that described in the subsection on RT-qPCR analysis of genes connected to apoptosis.

### 2.11. Ultraperformance Liquid Chromatography with Photodiode Array UV Detector and QDa Mass Spectrometry Conditions and Nuclear Magnetic Resonance (NMR) Analysis

The components of TFESP were separated by a Waters ACQUITY ultraperformance liquid chromatography (UPLC) system (Waters Corporation, Milford, MA, USA), which is equipped with a column compartment, a solvent manager system, an autosampler, and a photodiode array (PDA) and QDa mass spectrometry detectors. 1 *μ*L aliquot of the sample solution was injected into a CORTECS UPLC T3 column (1.6 *μ*m, 2.1 × 100 mm^2^) held at 40°C. The sampler temperature was kept at 25°C. The mobile phase was acetonitrile (A) and water with 0.1% (*v*/*v*) formic acid (B). The following optimized gradient program was used to separate and detect the components of TFESP: 22% A 3 min. The flow rate was 0.5 mL min^−1^ with a detection wavelength of 350 nm. Then, the TFESP was further purified by semipreparative HPLC. The chromatographic conditions were followed: the column Welchrom was a C18 column (10 × 250 mm^2^, 5 *μ*m) and the mobile phase was 22% acetonitrile while the flow rate was 2 mL min^−1^ and the detection wavelength was 246 nm with a column temperature of 25°C. The injection volume is 1 mL. The upper part of the highest peak of the half-peak width manually was collected with the continuous injection of TFESP. The collected liquid was combined and then dried at 70°C under reduced pressure to obtain a single compound which was then injected into UPLC for purity analysis. The obtained compound was dissolved in DMSO-d6 and analysed by 1H-NMR (600.13 MHz) and 13C-NMR (150.92 MHz) to infer the complete chemical structure.

### 2.12. Statistical Analysis

Data were shown as means ± standard error of the mean (SEM) of three independent experiments. The data were analysed using GraphPad Prism5 (GraphPad Software, San Diego, CA, USA). 3 randomly chosen samples per group are analysed by one-way ANOVA followed by LSD multiple comparison test.

## 3. Results and Discussion

### 3.1. Result

#### 3.1.1. In Vitro Antioxidant Assays

**Figure 1:**
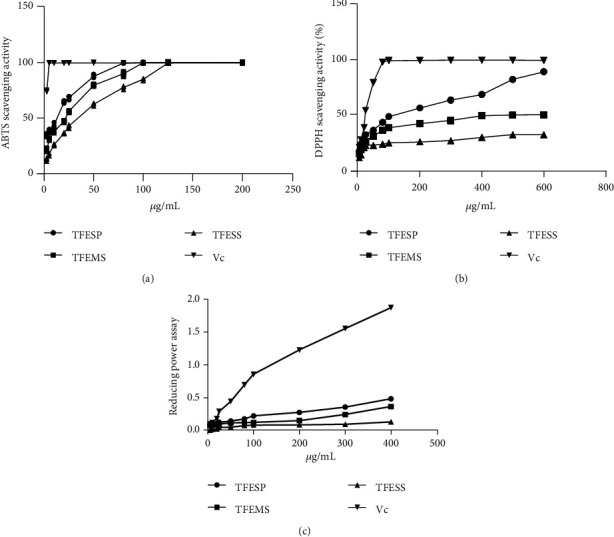
Antioxidant capacities of various extracts of saffron: (a) ABTS scavenging radical assay; (b) DPPH scavenging radical assay; (c) reducing power assay. Data are expressed as means ± standard error of the mean (SEM, *n* = 3). DPPH: 2,2-diphenyl-1-picrylhydrazyl; ABTS: 2,2′-azino-bis (3-ethylbenzothiazoline-6-sulfonic acid); Vc: vitamin C.

The TFC of TFESP was 602.1 mg RE/g, which was higher than that of TFESS and TFEMS (Table 1). Total flavonoid content is generally considered to be positively related to antioxidant activity. TFESP showed higher antioxidant activity than TFESS and TFEMS after the three antioxidant assays as shown in Figure 1. The IC_50_ of different extracts were determined using GraphPad as shown in Table 1. TFESP showed strong antioxidant activity because it contained high TFC. Therefore, it was further analysed in the following experiments.

#### 3.1.2. Protective Effects of TFESP against t-BHP-Induced Damage

**Figure 2:**
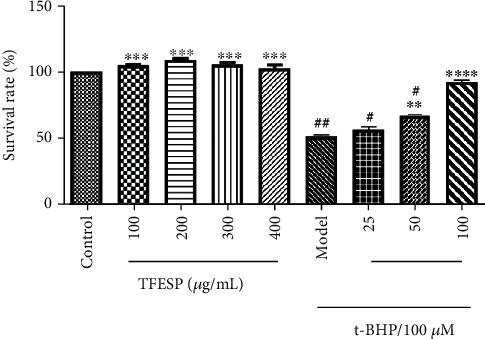
Cytotoxicity and cytoprotective effects of total flavonoid extract from saffron petals (TFESP). BRL-3A cells treated with various concentrations of TFESP (50–300 *μ*g/mL) for 24 h and pretreated for 24 h with indicated TFESP concentrations (low concentration, 25 *μ*g/mL; middle concentration, 50 *μ*g/mL; high concentration, 100 *μ*g/mL) before treatment with 100 *μ*M t-BHP. Statistical significance: ^#^*p* < 0.05, ^##^*p* < 0.01, ^###^*p* < 0.001, and ^####^*p* < 0.0001, contrasted to the control group; ^∗^*p* < 0.05, ^∗∗^*p* < 0.01, ^∗∗∗^*p* < 0.001, and ^∗∗∗∗^*p* < 0.0001, contrasted to the t-BHP-treated group.

After being treated with 50–300 *μ*g/mL TFESP for 24 h, the viabilities of BRL-3A cells ranged from 100.000% to 108.897%, which indicated that there was no obvious difference between the TFESP-treated group and the blank control group (Figure 2). However, after treatment with only t-BHP, the cell viability was markedly decreased contrasted to that of the control group. After 24 h pretreatment with TFESP (25, 50, and 100 *μ*g/mL), the BRL-3A cells were exposed to 100 *μ*M t-BHP for 2 h, and their survival rates were markedly higher than those of the cells without pretreatment with TFESP. Moreover, these changes showed concentration-dependence. Therefore, TFESP protected BRL-3A cells from t-BHP-induced oxidative stress.

#### 3.1.3. TFESP Decreased Accumulation of ROS in BRL-3A Cells

**Figure 3:**
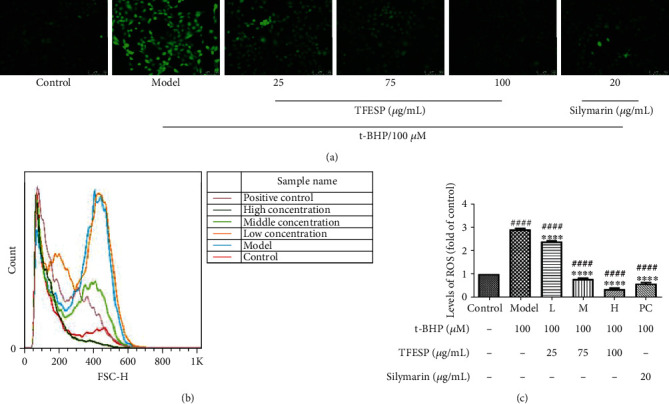
Total flavonoid extract from saffron petals (TFESP) restrains reactive oxygen species (ROS). Fluorescence of BRL-3A cells: (a) observed under fluorescence microscope (100x magnification); (b) detected by flow cytometry; (c) quantified. Statistical significance: ^#^*p* < 0.05, ^##^*p* < 0.01, ^###^*p* < 0.001, and ^####^*p* < 0.0001, contrasted to the control group; ^∗^*p* < 0.05, ^∗∗^*p* < 0.01, ^∗∗∗^*p* < 0.001, and ^∗∗∗∗^*p* < 0.0001, contrasted to the t-BHP-treated group.

ROS levels in the cells were detected by a DCFH-DA kit. The pretreatment with TFESP remarkably reduced t-BHP-induced ROS amassing in a dose-dependent manner, and the effect was even better than silymarin (Figure 3(a)). The result of flow cytometry indicated that the intracellular ROS rising by threefold in the t-BHP-treated cells contrasted with the untreated control (Figure 3(c)) while intracellular ROS was remarkably reduced after pretreatment by TFESP. And the ROS levels in BRL-3A cells pretreated with 100 *μ*g/mL TFESP were even lower than those of the control cells that were not treated with t-BHP or not pretreated with silymarin (Figures 3(b) and 3(c)). These results indicated that TFESP inhibited t-BHP-induced intracellular ROS accumulation.

#### 3.1.4. TFESP Inhibited t-BHP-Induced Apoptosis

**Figure 4:**
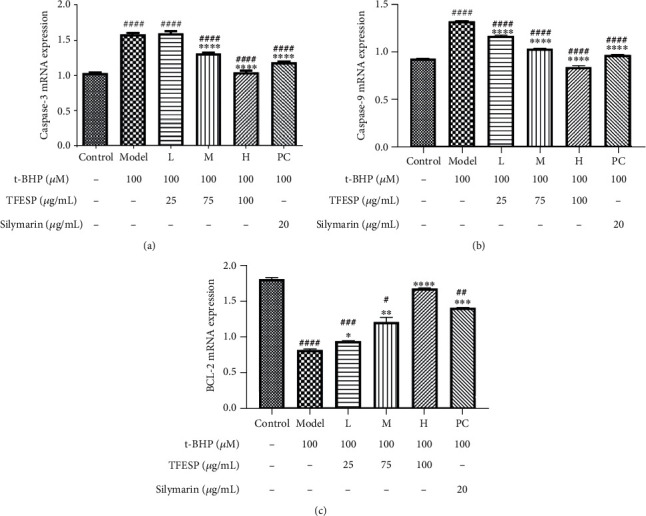
RT-qPCR analysis of expression of apoptosis-related genes. (a) Effect of TFESP on expression of *caspase-3*. (b) Effect of TFESP on expression of *caspase-9*. (c) Effect of TFESP on expression of *Bcl-2*. RT-qPCR: quantitative real-time reverse transcription-polymerase chain reaction. Statistical significance: ^#^*p* < 0.05, ^##^*p* < 0.01, ^###^*p* < 0.001, and ^####^*p* < 0.0001, contrasted to the control group; ^∗^*p* < 0.05, ^∗∗^*p* < 0.01, ^∗∗∗^*p* < 0.001, and ^∗∗∗∗^*p* < 0.0001, contrasted to the t-BHP-treated group.

The pretreatment with specific TFESP concentrations prevented t-BHP-induced apoptosis, which is even better than the positive control. Therefore, the protective effect on the BRL-3A cell of TFESP against t-BHP was antiapoptotic. The expression levels of some pivotal genes relevant to apoptosis were determined. As shown in Figures 4(a)–4(c), the expression of *B-cell lymphoma-2* (*Bcl-2*) was reduced while those of *caspase-3* and *caspase-9* were raised after the cells were treated by t-BHP. Therefore, with pretreatment of TFESP or silymarin, t-BHP-induced apoptosis in BRL-3A cells was repressed by downregulating the expressions of *caspase-3* and *caspase-9* and causing the expressions of the antiapoptotic gene *BCL-2*. These results indicated that TFESP could reduce oxidative stress induced by t-BHP in BRL-3A cells via the antiapoptosis pathway.

#### 3.1.5. Key Enzyme Activities in Cell Supernatants

**Figure 5:**
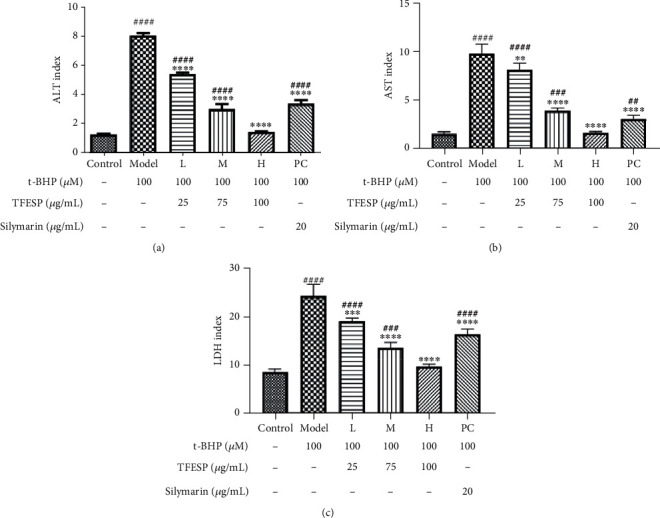
Activities of crucial enzyme existed in cell supernatant: (a) ALT; (b) AST; (c) LDH. ALT: alanine aminotransferase; AST: aspartate transaminase; LDH: lactate dehydrogenase. Statistical significance: ^#^*p* < 0.05, ^##^*p* < 0.01, ^###^*p* < 0.001, and ^####^*p* < 0.0001, contrasted to the control group; ^∗^*p* < 0.05, ^∗∗^*p* < 0.01, ^∗∗∗^*p* < 0.001, and ^∗∗∗∗^*p* < 0.0001, contrasted to the t-BHP-treated group.

The changes of ALT and AST activities can partly reflect the health status of the liver. In this study, the activities of ALT and AST in BRL-3A cells treated with t-BHP were remarkably higher than those of the relevant control groups. However, TFESP pretreatment effectively inhibited the increases of ALT (Figure 5(a)) and AST (Figure 5(b)) in t-BHP-treated BRL-3A cells with concentration-dependence, and the inhibition rate is higher than that of silymarin. Intracellular LDH induced by t-BHP in the cells pretreated with TFESP was also reduced (Figure 5(c)) in contrast to that of cells without pretreatment with TFESP.

#### 3.1.6. Protective Effects of TFESP on Antioxidant Activities

**Figure 6:**
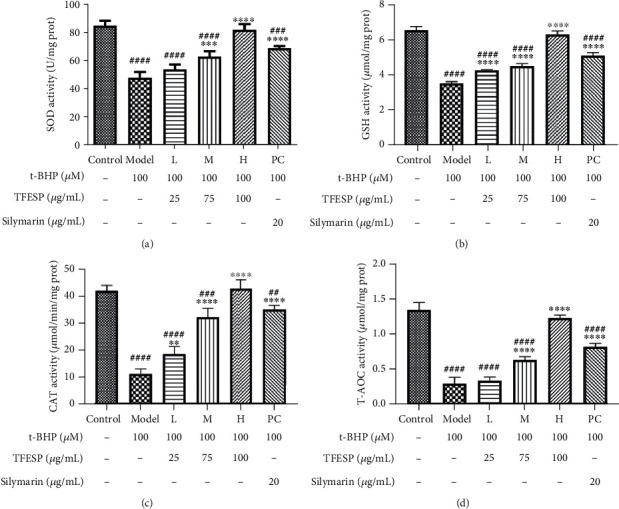
Effect of total flavonoid extract from saffron petals (TFESP) on antioxidant enzymes or substance: (a) SOD; (b) GSH; (c) CAT; (d) T-AOC. SOD: superoxide dismutase; GSH: glutathione; CAT: catalase; T-AOC: total antioxidant capacity. Statistical significance: ^#^*p* < 0.05, ^##^*p* < 0.01, ^###^*p* < 0.001, and ^####^*p* < 0.0001, contrasted to the control group; ^∗^*p* < 0.05, ^∗∗^*p* < 0.01, ^∗∗∗^*p* < 0.001, and ^∗∗∗∗^*p* < 0.0001, contrasted to the t-BHP-treated group.

SOD (Figure 6(a)), CAT (Figure 6(c)), and TAOC (Figure 6(d)) activity was remarkably reduced in the t-BHP treatment group compared with the control group. The activity of these enzymes of cells pretreated with TFESP was remarkably raised, better than silymarin. The level of GSH in the cells treated with TFESP was remarkably raised, which enhanced antioxidant capacity as shown in Figure 6(b). Consequently, TFESP may increase the antioxidant enzyme activities and GSH levels to defend the cells against oxidative damage induced by t-BHP. After analysing the antioxidant pathway and the relative expression of part related genes, the protective effect of TFESP on BRL-3A cells subjected to t-BHP-induced oxidative stress was explored.

#### 3.1.7. Effects of TFESP on Expression of Antioxidant Genes

**Figure 7:**
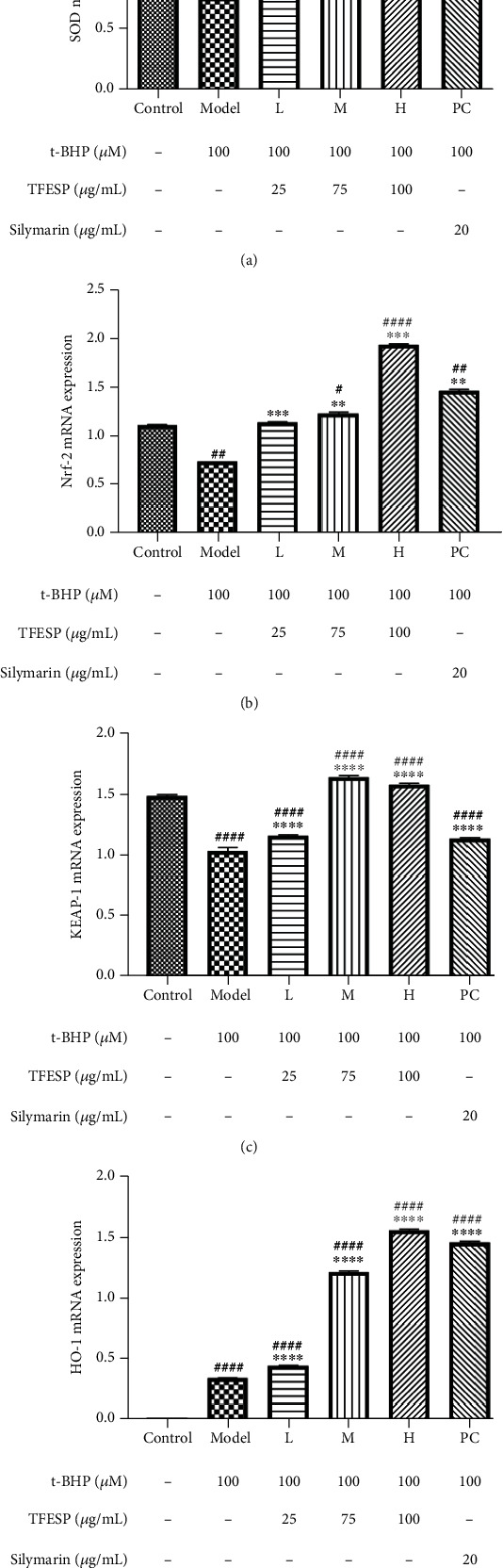
Expression of gene related to antioxidants: (a) *SOD*; (b) *Nrf2*; (c) *KEAP-1*; (d) *HO-1*. *SOD*: *superoxide dismutase*; *Nrf2*: *nuclear factor, erythroid 2-related factor 2*; *KEAP-1*: *Kelch-like ECH-associated protein-1*; *HO-1*: *heme oxygenase 1*. Statistical significance: ^#^*p* < 0.05, ^##^*p* < 0.01, ^###^*p* < 0.001, and ^####^*p* < 0.0001, contrasted to the control group; ^∗^*p* < 0.05, ^∗∗^*p* < 0.01, ^∗∗∗^*p* < 0.001, and ^∗∗∗∗^*p* < 0.0001, contrasted to the t-BHP-treated group.

Oxidative stress can affect the expression level of antioxidant genes. When ROS production exceeds the threshold controlled by the antioxidant defense system, many diseases will occur one after another. The RT-qPCR analysis (Figure 7) indicated that t-BHP strongly restrained the expression of *SOD* mRNA. When cells were pretreated by TFESP, t-BHP-treated cells expressed *SOD* mRNA normally. When cells were challenged with t-BHP, some antioxidant genes (*SOD*, *HO-1*, *Keap-1*, and *Nrf2*) were remarkably decreased, while the expression of antioxidant genes of cells pretreated with TFESP showed a remarkable rise compared with the model group cells (Figure 7). Furthermore, the above effects of TFESP are better than those of silymarin. Therefore, TFESP prevented cell damage, which was induced by intracellular oxidative stress. Clearly, TFESP could increase the expression of antioxidant genes to improve the antioxidant capacity of cells.

#### 3.1.8. Effects of TFESP on Expression of Inflammation Genes

**Figure 8:**
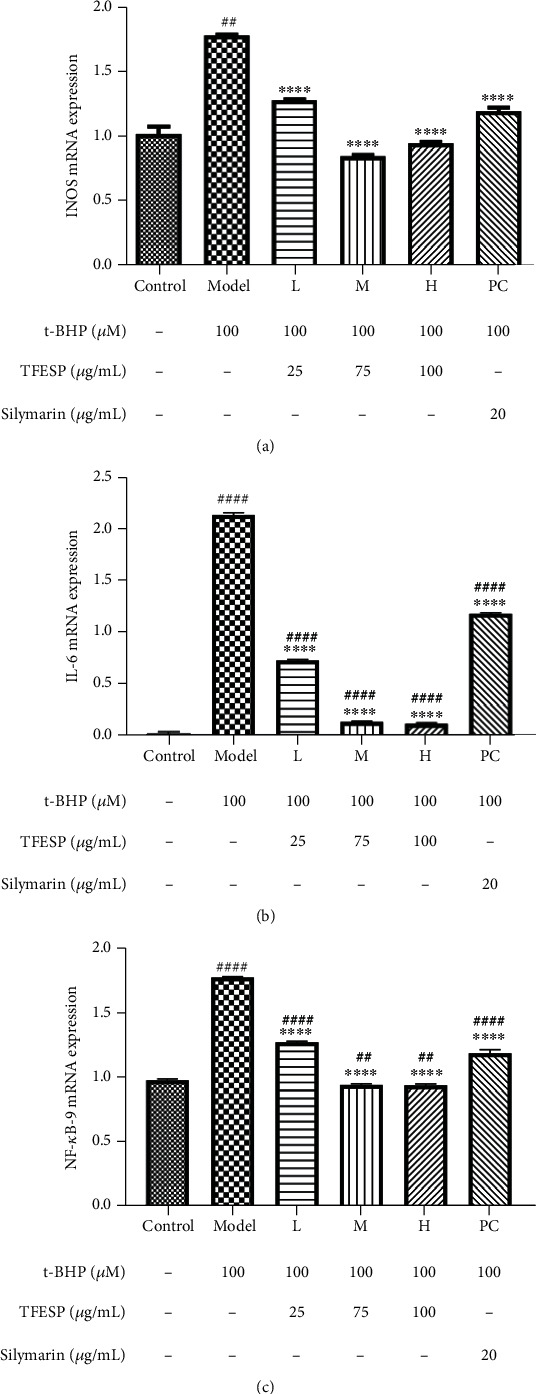
Expression of inflammation-related genes: (a) effect of TFESP on expression of *INOS*; (b) effect of TFESP on expression of *IL-6*; (c) effect of TFESP on expression of *NF-κB-9*. *INOS*: *inducible nitric oxide synthase*; *IL-6*: *interleukin-6*; *NF-κB-9*: *nuclear factor kappa B-9*. Statistical significance: ^#^*p* < 0.05, ^##^*p* < 0.01, ^###^*p* < 0.001, and ^####^*p* < 0.0001, contrasted to the control group; ^∗^*p* < 0.05, ^∗∗^*p* < 0.01, ^∗∗∗^*p* < 0.001, and ^∗∗∗∗^*p* < 0.0001, contrasted to the t-BHP-treated group.

The expression of inflammation genes such as *INOS*, *IL-6*, and *NF-κB-9* was analysed, and the protective effect of TFESP on t-BHP-induced inflammation of BRL-3A cells was investigated. Our results showed that the expression levels of *INOS* (Figure 8(a)), *IL-6* (Figure 8(b)), and *NF-κB-9* (Figure 8(c)) were obviously upregulated when the BRL-3A cells were treated with t-BHP. However, the expression of anti-inflammatory-related genes in cells pretreated with TFESP was notably reduced, and it was concentration-dependent. These results revealed that TFESP protects cells from damage by attenuating the expression of inflammation-related genes.

#### 3.1.9. UPLC-PDA/MS and NMR Analysis of TFESP

**Figure 9:**
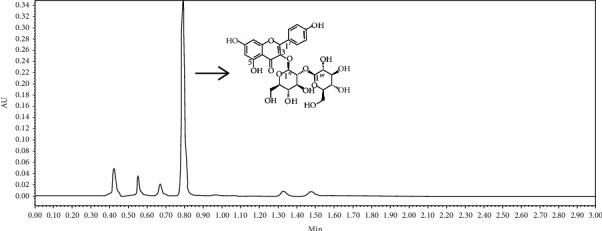
Quantitative analysis and qualitative analysis of TFESP.

The main component (85%) of TFESP was analysed by UPLC as a flavonoid compound with a relative molecular mass of 610 (Figure 9). After comparison with the data in the relevant literature [24, 25], it was preliminarily speculated that this monomer compound might be one of the following three kaempferol glucosides: kaempferol-3,4′-di-o-glucoside, kaempferol-3,7-di-o-glucoside, and kaempferol-3-o-sophoroside.

The results from nuclear magnetic analysis were as follows: 1H − NMR (600.13 MHz, DMSO − D6) = 8.04, 8.04, 6.91, 6.91, 6.42, 6.19, 5.70 (*J* = 6.6 Hz), 4.61 (*J* = 7.6 Hz), 3.60, 3.51, 3.48, 3.47, 3.40, 3.28, 3.28, 3.20, 3.18, 3.15, 3.13, and 3.10; 13C-NMR (150.92 MHz, DMSO-d6) are 177.88, 164.85, 161.70, 160.39, 156.78, 155.97, 133.30, 131.37, 131.37, 121.36, 115.73, 115.73, 104.55, 104.28, 99.19, 98.41, 94.08, 82.90, 77.93, 77.46, 77.07, 77.03, 74.85, 70.18, 70.04, 61.29, and 61.00. The NMR data of kaophenol-3-o-sophora were basically consistent with those reported previously [25, 26]. Therefore, the isolated flavonoid monomer was characterized as kaempferol-3-o-sophoroside (KOS), and its chemical structure is shown in Figure 9.

#### 3.1.10. Monomer Activity Verification

**Figure 10:**
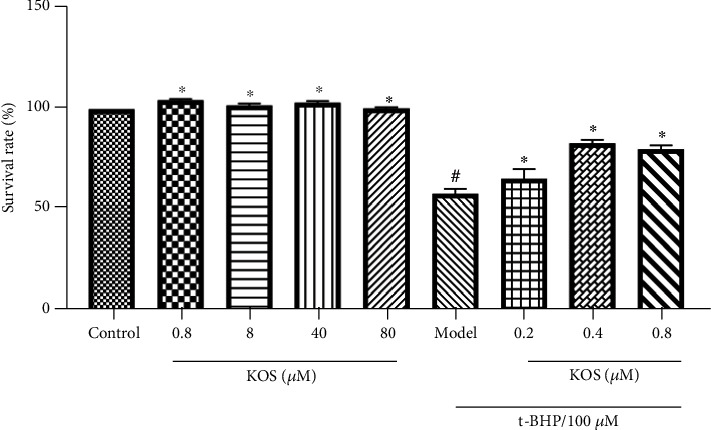
Cytotoxicity and cytoprotective effects of kaempferol-3-o-sophorin (KOS). Control: BRL-3A cells exposed to various concentrations of KOS (0.8, 8, 40, and 80 *μ*M) for 24 h and pretreated for 24 h with indicated KOS concentrations before treatment with 100 *μ*M t-BHP. Statistical significance: ^#^*p* < 0.05, ^##^*p* < 0.01, ^###^*p* < 0.001, and ^####^*p* < 0.0001, contrasted to the control group; ^∗^*p* < 0.05, ^∗∗^*p* < 0.01, ^∗∗∗^*p* < 0.001, and ^∗∗∗∗^*p* < 0.0001, contrasted to the t-BHP-treated group.

After treatment with only t-BHP, the cell viability was noteworthily decreased contrasted with that of the control group. After 24 h pretreatment with KOS (0.2, 0.4, and 0.8 *μ*M), the BRL-3A cells were exposed to 100 *μ*M t-BHP for 2 h, and their survival rates were markedly higher than those of the untreated cells. Furthermore, these changes were concentration-dependent. When treated with 0.8–80 *μ*M KOS for 24 h, the BRL-3A cells had viabilities of 99.870%–104.91% (Figure 10), which were not obviously different from the control group (*p* > 0.05).

### 3.2. Discussion

The ROS is a conventional product of normal oxygen metabolism in the human body, usually maintained at a relatively stable level under normal circumstances [27]. However, when the body is affected by factors, such as mental stress, environmental pollution, or drugs, the balance of oxygen metabolism will be broken. It follows that ROS will be produced in large quantities and accumulated in the body and then oxidative stress will occur, which causes various pathological changes [28], such as apoptosis, inflammation, and liver disease. Therefore, the ROS level is an important indicator of the body's oxygen metabolism level. The medicinal plants with high levels of flavonoids [29] and phenolic compounds [30] have been showed to be capable of effectively preventing liver diseases caused by oxidative stress through directly scavenging ROS and indirectly affecting the antioxidant defense system.

Investigating the antioxidant and hepatoprotective activities of saffron petals showed that TFESP contained a high total flavonoid content of 602.1 mg RE/g, and had stronger scavenging ability on ABTS and DPPH than that of TFESS and TFEMS. In the hepatoprotective test, TFESP showed no cytotoxicity when concentration reached 300 *μ*g/mL, and TFESP was able to restore t-BHP-induced liver damage in a concentration-dependent manner.

These results further showed that TFESP can effectively reduce the accumulation of ROS in hepatocytes induced by t-BHP, and even the intracellular ROS levels in the high-dose group were lower than those in the control and positive controls. The previous studies have found that the *Keap-1*/*nrf2-ARE-HO-1* antioxidant pathway is a common cellular antioxidant pathway [31]. Our results of the RT-qPCR test demonstrated that TFESP could enhance the expression of this antioxidation pathway-related gene (*Keap-1* and *Nrf2*, *HO-1*) and the upstream gene p38 in hepatocytes. And TFESP could also increase SOD mRNA in hepatocytes. From this point of view, TFESP may increase the antioxidant capacity of hepatocytes by upregulating the genes related to the antioxidation pathway of *Keap-1/nrf2-ARE-HO-1*, thereby being able to cope with the oxidative stress caused by t-BHP-induced ROS accumulation. Interestingly, the ability of TFESP upregulation on the above genes is more efficient than silymarin, a kind of liver-protecting medicine. This suggests that the petals of saffron have the potential of being developed into liver-protecting drugs, which not only expands the resources of liver-protecting drugs but also recycles the biowaste of saffron.

Based on this finding, we investigated the antioxidant-related enzyme activities and total antioxidant capacity of hepatocytes. It is well known that GSH can effectively reduce free radical intermediates and peroxides [32], thereby exerting an antioxidant effect. SOD and CAT play a role in the conversion of H_2_O_2_ and O^2-^, and H_2_O, respectively [33]. Our testing the activity of SOD, GSH, and CAT, as well as the total antioxidant capacity of T-AOC, showed that TFESP could effectively enhance SOD, GSH, and CAT activities and enhance total antioxidant capacity.

Inflammation and apoptosis [34] are responsible for cell death in the body, Therefore, the expression of inflammation-related genes such as *INOS*, *IL-6*, and *NF-κB-9* [35], which are closely related to the occurrence of inflammation, was analysed to understand the relationship between hepatoprotective activity of TFESP and intracellular inflammation. TFESP reversed the upregulation of the t-BHP-induced expression of *INOS*, *IL-6*, and *NF-κB-9*. Therefore, TFESP protects cells from damage by attenuating the expression of inflammation-related genes. Simultaneously, we considered the effect of TFESP on apoptosis. Many studies have shown that *caspase-3* and *caspase-9* [36] are apoptosis-related genes, while *BCL-2* [37] is an antiapoptotic gene. In this study, we also measured the expression levels of these three genes in each group by RT-qPCR and found that TFESP can reverse the upregulation of the *caspase-3* and *caspase-9* expression and the downregulation of the *BCL-2* expression in hepatocytes induced by t-BHP. It indicates that TFESP can reverse the t-BHP-induced apoptosis of hepatocytes.

Previous studies had shown that kaempferol [38] and its glycoside compounds [39] have hepatoprotective activity. In this study, our analysis of TFESP using UPLC-PDA/MS revealed that kaempferol-3-o-sophoroside (a glycosidic compound of kaempferol) is the main component of TFESP. Therefore, the hepatoprotective activity of TFESP may be attributed to the large amount of kaempferol-3-o-sophoroside.

The monomer activity verification of the kaempferol-3-o-sophorin monomer showed that it has a significant cytoprotective effect. Therefore, we speculate that KOS is the key substance for TFESP to protect BRL-3A cells against t-BHP-induced oxidative stress. The optimal effective concentration of TFESP to inhibit t-BHP-induced oxidative damage was 100 mg/mL, while the monomer KOS was 0.48 *μ*g/mL (0.8 *μ*mol). The effect of the monomer is much better than that of TFESP. The reason may be that other substances in the mixture affect the activity of KOS. The studies have shown that TFESP and kaophenol-3-o-sophora are a safe and promising hepatoprotective agent.

Existing studies have shown that the petals of saffron are rich in polyphenols, flavonoids, and polysaccharides [40]. These substances, such as polyphenols and polysaccharides, also have more important activities, but some are not good for the health. Rao et al. have shown that the addition of saffron on heavy metal chromium by cadmium stress experiments of saffron found that saffron root, bulb, stigma, stamen, and petal parts have the ability to enrich heavy metals. The enrichment ability of the root is the strongest while the ability of petals is the weakest [41]. But at present, there are no reports related to saffron products of overweight heavy metals or metal toxicity. It is interesting that a study shows that the petals are rich in a variety of metal elements suitable as a feed [42]. Considering that saffron does indeed have the ability to enrich heavy metals, it is necessary to consider the detection of heavy metals in the product and the selection of suitable soil when planting saffron, especially when it is used as food, health products, or medicine.

## 4. Conclusions

Petals of saffron have high total flavonoid content. We showed that TFESP has hepatoprotective potential due to its rich total flavonoid content and strong antioxidant activity. TFESP may effectively reduce the accumulation of ROS in response to oxidative stress caused by t-BHP. Furthermore, TFESP effectively protected BRL-3A cells from being damaged by *t-BHP* through increasing the expression of antiapoptotic genes (*BCL-2*), counteracting oxidative stress-related enzymes or genes (*SOD*, *CAT*, *GSH* and *T-AOC*, *Keap-1*, *Nrf2*, *HO-1*, and *SOD* mRNA). Moreover, TFESP may effectively downregulate the expression of apoptosis-related genes *caspase-3* and *caspase-9*, the inflammation-related genes *iNOS* and *IL-6*, and *NF-κB-9*. In other words, TFESP can enhance the antioxidative capacity, antiapoptotic ability, and anti-inflammatory ability of BRL-3A cells to cope with oxidative stress damage induced by t-BHP. The biological activity of plants is mostly related to the chemical components contained in plants. Further analysis revealed that TFESP contains a variety of chemicals; the main chemical composition is kaempferol-3-o-sophoroside. The protective activity of TFESP on injured BRL-3A cells ought to be attributed to kaempferol-3-o-sophoroside. Although how kaempferol-3-o-sophoroside plays a protective role needs to be further investigated, we believe that the biowaste of saffron petals has good development and application prospects in health care products and medicines for liver protection.

## Figures and Tables

**Table 1:** Flavonoid content and antioxidant activities of various extracts of saffron.

Sample	Flavonoid content (mg RE/g)	IC_50_ (*μ*g/mL)	
		DPPH	ABTS
TFESP	602.1 ± 1.960	94.08 ± 1.072	8.844 ± 1.096
TFEMS	153.033 ± 1.109	485.2 ± 5.541	15.37 ± 1.082
TFESS	66.167 ± 1.676	18593 ± 1.329	27.95 ± 1.060
Vc	nd	21.1 ± 1.048	2.115 ± 1.027

TFESP: total flavonoid extract from saffron petals; TFESS: total flavonoid extract from saffron stamens; TFEMS: total flavonoid extract from mixture of saffron petals and stamens; DPPH: 2,2-diphenyl-1-picrylhydrazyl; ABTS: 2,2′-azino-bis (3-ethylbenzothiazoline-6-sulfonic acid); nd: not detected.

## Data Availability

The primer sequences used in the RT-qPCR assay were all included in the supplemental material (S-Table 1). GAPDH is used as an internal reference gene. The abbreviation used in this study is listed in the supplemental material (S-Table 2).

## Supplementary Materials

Supplementary Materials S-Table 1: primer sequences. S-Table 2: abbreviation.
