# Supplementary material for: Total Flavonoids of Crocus sativus Petals Release tert-Butyl Hydroperoxide-Induced Oxidative Stress in BRL-3A Cells
Source: Oxid Med Cell Longev. 2021 Jun 5;2021:5453047. doi: 10.1155/2021/5453047 (PMC8203408; doi:10.1155/2021/5453047)
Supplement: Supplementary Materials — S-Table 1: primer sequences. S-Table 2: abbreviation. [file 5453047.f1.doc]

S-table 1. Primer sequences

| Primer Nucleotide Sequences | | | Annealing  Temp (°C) |
| --- | --- | --- | --- |
| *Caspase-3* | For. 5’: | GGATGGGTGCTATTGTGAGG | 60 |
|  | Rev. 5’: | TGGGATTTCAAGGCGACG |  |
| *Caspase-9* | For. 5’: | CTTGTGTCCTACTCCACCTTC | 58 |
|  | Rev. 5’: | GTTAAAACAGCCAGGAATCTGC |  |
| *Bcl-2* | For. 5’: | GTGGATGACTGAGTACCTGAAC | 60 |
|  | Rev. 5’: | CTTCACTTGTGGCCCAGATAG |  |
| *INOS* | For. 5’: | GACCAAACTGTGTGCCTGGA | 56 |
|  | Rev. 5’: | TACTCTGAGGGCTGACACAAGG |  |
| *IL-6* | For. 5’: | CTGGAGTTCCGTTTCTACCTG | 56 |
|  | Rev. 5’: | CCTTCTGTGACTCTAACTTCTCC |  |
| *NF-kB-9* | For. 5’: | CTACGAGACCTTCAAGAGCATC | 58 |
|  | Rev. 5’: | GATGTTGAAAAGGCATAGGGC |  |
| *P38* | For. 5’: | ATATTTGGTCCGTGGGCTG | 56 |
|  | Rev. 5’: | AGTTCATCTTCGGCATCTGG |  |
| *HO-1* | For. 5’: | CTTTCAGAAGGGTCAGGTGTC | 58 |
|  | Rev. 5’: | TGCTTGTTTCGCTCTATCTCC |  |
| *Nrf2* | For. 5’: | CAGAAGGAACAGGAGAAGGC | 58 |
|  | Rev. 5’: | GCATACAGTCTTCAAAGTACAAGG |  |
| *SOD* | For. 5’: | GGACAAACCTGAGCCCTAAG | 60 |
|  | Rev. 5’: | GCAATCTGTAAGCGACCTTG |  |
| *GAPDH* | For. 5’: | AACGACCCCTTCATTGACC | 60 |
|  | Rev. 5’: | CACGACATACTCAGCACCAG |  |

1. table 2. Contractions

| Abbreviation | Full name | Abbreviation | Full name |
| --- | --- | --- | --- |
| ABTS | 2，2ʹ-azino-bis (3-ethylbenzthiazoline-6-sulfonic acid) | MS | mass spectrometry |
| ALT | Alanine aminotransferase | *NF-KB-9* | nuclear factor-kb-9 |
| AST | aspartate transaminase | *Nrf2* | nuclear factor, erythroid 2-related factor 2 |
| BCA | bicinchoninic acid | PDA | photodiode array |
| Bcl-2 | B-cell lymphoma-2 | RT-*q*PCR | Real-timequantitative PCR |
| CCK-8 | Cell counting kit 8 | RIPA | Radio immunoprecipitation assay |
| DAPI | 4，6-Diamidino-2-phenylindole | SOD | superoxide dismutase |
| DCFH-DA | 2',7'-dichlorodihydrofluorescein diacetate | T-AOC | total antioxidant capacity |
| DPPH | 1,1-diphenyl-2-picrylhydrazyl | *t*-BHP | Tert-butyl hydroperoxide |
| GAPDH | Glyceraldehyde 3-phosphate dehydrogenase | TFESP | total flavonoid extracts of saffron petals |
| GSH | glutathione | TFESS | total flavonoid extracts of saffron stamens |
| *HO-1* | heme oxygenase 1 | TFEMS | total flavonoid extracts of saffron petals and stamens |
| IL-6 | interleukin-6 | Vc | Vitamin C |
| INOS | inducible nitric oxide synthase | UPLC | ultrahigh performance liquid chromatography |
| *Keap-1* | Kelch-like ECH-associated protein-1 | UPLC | ultrahigh performance liquid chromatography |
| LDH | lactate dehydrogenase |  |  |
